# A Rare Case of Checkerboard-Like Becker Nevi with a Unique Distribution of Abnormalities

**DOI:** 10.1155/2019/2387365

**Published:** 2019-05-14

**Authors:** Chanidapa Wongtada, Pravit Asawanonda, Marisa Pongprutthipan

**Affiliations:** ^1^M.D., Graduated from Chulalongkorn University, Bangkok, Thailand; ^2^Division of Dermatology, Department of Medicine, King Chulalongkorn Memorial Hospital, Bangkok, Thailand

## Abstract

Becker nevus syndrome refers to a rare disorder comprising the typical pigmented lesion and its associated developmental abnormalities. Becker nevus itself is typically localized on the upper trunk, scapular or upper arm unilaterally; however, it can occasionally occur as multiple or bilateral lesions on any parts of the body. In this report, a rare case of multiple Becker nevi arranged in a unique checkerboard-like pattern is presented. More uniquely, the associated abnormalities found in this patient, breast hypoplasia and leg lipodystrophy, seem to be regionally correlated with the nevi. In closing, we would like to raise an easy-to-remember 6B's mnemonic, which stands for Becker, Breast, Bone, Bowen's disease, Basal cell carcinoma, and Beta-actin, for the cases of Becker nevus syndrome.

## 1. Introduction

Becker nevus is a well-defined, irregular border, tan to dark brown hyperpigmented cutaneous hamartoma, which is commonly associated with hypertrichosis and presented in unilateral localized fashion on upper trunk. Less common sites are extremities, face/neck, abdomen, and lower back, respectively [1]. There are various malformations that are in association with Becker nevi, contributing to Becker nevus syndrome. The common associated malformations include breast hypoplasia, musculoskeletal malformation, and scoliosis [1]. Interestingly, according to a systematic literature review by Schneider et al., Becker nevi and the malformations seem to have a regional correspondence [1]. In this case report, we present an uncommon case of Becker nevus syndrome with a unique checkerboard mosaic pattern and regional corresponding breast hypoplasia and leg lipodystrophy.

## 2. Case Report

**Figure 1:**
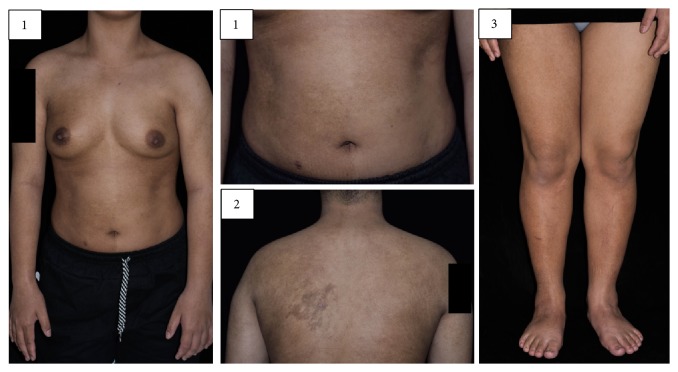
Multiple Becker nevi on (1) anterior trunk resembling a checkerboard mosaic pattern, (2) left upper back extending toward left shoulder, and (3) the whole right leg. Slight smaller left breast and right leg circumference are seen.

A 20-year-old woman presented with multiple hyperpigmented patches involving the entire right hemibody and left upper back since birth. The patches were asymptomatic and became darker during puberty. There was no family history of similar disorders. On skin examination, multiple well-defined, irregular-border hyperpigmented patches were observed on the entire right side of the body with alternate areas of pigmentary change and sharp demarcation at anterior midline, resembling a checkerboard mosaic pattern. Moreover, there were dark brown patches on left upper back extending towards left shoulder and left chest. Further examination showed left breast hypoplasia and a slight smaller circumference of right leg comparing to the left (Figure 1). Ultrasonography of breasts confirmed relatively small size of left breast with normal appearance of fibroglandular tissue. Plain-film X-rays of both legs found no bony abnormalities with an equal leg length and similar shadow of muscle masses. Considering this evidence, the smaller right leg most likely results from subcutaneous fat hypoplasia.

On histopathology of the skin biopsy from left upper back and right abdomen, both sites exhibited acanthosis, slightly increased number of melanocytes along the basal layer of epidermis, mild elongation, and bridging of hyperpigmented rete ridges, compatible with Becker nevus.

## 3. Discussion

Although most Becker nevi are found as isolated findings, some can be associated with abnormalities. Their precise etiopathogenesis still remains unclear. However, recent findings demonstrated that postzygotic lethal mutations in ACTB gene, a gene coding for beta-actin, may underlie the development of both Becker nevus and Becker nevus syndrome [2]. It has been hypothesized that in Becker nevus syndrome this sporadic mutation might occur during early developmental period, thus affecting multiple cell lineages compared with an isolated Becker nevus [2]. Androgen-dependent mechanism is another proposed pathogenesis of the disease according to its onset during puberty, the presence of hypertrichosis, and acneiform eruption [3], as well as the increase in androgen receptors restricted to the involved region [4].

According to a systematic literature review by Schneider et al., there is a regional correspondence between the skin manifestation of Becker nevus and the malformation, providing a clue that there might be a common mutation during embryogenesis. Breast hypoplasia, maxillofacial dysplasia, and lipodystrophy were reported to have the strongest regional correspondence [1]. In this patient, we present an interesting case of Becker nevus syndrome with checkerboard mosaic skin pattern with left breast hypoplasia and right leg lipodystrophy. Apparently, these two abnormalities are correlated with the sites of Becker nevi. Hence, this case is an excellent example showing the regional correspondence and raising awareness that Becker nevus may accompany some abnormalities, so careful examination should be performed.

Checkerboard or flag-like or block-like pattern is one of the archetypical patterns, which is characterized by alternating squares with a sharp midline separation. It can be found in either cases of cutaneous mosaicism or genetic chimerism. In case of mosaicism, if the mutation occurred early in embryogenesis, it is more likely that abnormal clone will be widely spread, contributing to a larger number of skin lesions and multiorgan involvement [5]. As a result, checkerboard skin pattern, which consists of multiple skin lesions, is sometimes reported to co-occur with extracutaneous abnormalities with frequent topographic correspondence between the skin lesions and the tissues involved. It is occasionally found in a number of different genetically determined syndromes [6]. Though multiple Becker nevi in one patient can be found from time to time, only few exhibit the real checkerboard configuration, emphasizing that checkerboard-like skin pattern is considered a rare condition among cases of Becker nevi or Becker nevus syndrome. Up to present, from our review, only three cases have been reported with multiple Becker nevi distributed in checkerboard configuration and none of them coexists with regional correspondence abnormalities like in our case.[7, 8]

The nature of Becker melanosis is benign; however, three reports have revealed that it might be associated with keratinocyte carcinomas such as basal cell carcinoma and Bowen disease. All of three cases developed cancerous lesions over the area of Becker nevus, which was a sun-protected and atypical site in a low-risk patient [9, 10]. Though it might be coincidental, awareness should be raised about long-term malignancy development in Becker nevus. We recommend a thorough and regular long-term follow-up in both cases of Becker nevus and Becker nevus syndrome. Especially in cases with leg dysplasia, long-term monitoring for joint deformities is warranted. Last but not least, from all the discussion so far, we would like to propose 3 more B's; Bowen disease, Basal cell carcinoma, and Beta-actin to the already well-known Becker, Breast, and Bone as an easy mnemonic for Becker nevus syndrome.

In conclusion, we present a case of Becker nevus syndrome which has very unique and interesting features. It is one of few cases that reveal a checkerboard mosaic pattern of Becker nevi and apparently point up the correlation between sites of Becker nevi and abnormalities involved. Moreover, we strengthen the point that long-term follow-up should be kept in mind for upcoming associated abnormalities, complication, and malignancy.
